# Association between Tooth Loss and Cognitive Function among 3063 Chinese Older Adults: A Community-Based Study

**DOI:** 10.1371/journal.pone.0120986

**Published:** 2015-03-24

**Authors:** Jianfeng Luo, Bei Wu, Qianhua Zhao, Qihao Guo, Haijiao Meng, Lirong Yu, Li Zheng, Zhen Hong, Ding Ding

**Affiliations:** 1 Department of Health Statistics and Social Medicine, School of Public Health, Fudan University, The Key Laboratory of Public Health Safety of Ministry of Education, Shanghai, China; 2 School of Nursing and Global Health Institute, Duke University, Durham, North Carolina, United States of America; 3 Institute of Neurology, Huashan Hospital, Fudan University, WHO Collaborating Center for Research and Training in Neurosciences, Shanghai, China; 4 School of Sociology and Political Sciences, Shanghai University, Shanghai, China; UNC School of Dentistry, University of North Carolina-Chapel Hill, UNITED STATES

## Abstract

**Background:**

Oral health has been found to be associated with cognitive function in basic research and epidemiology studies. Most of these studies had no comprehensive clinical diagnosis on cognitive function. This study firstly reported the association between tooth loss and cognitive function among Chinese older population.

**Methods:**

The study included 3,063 community dwelling older adults aged 60 or above from the Shanghai Aging Study. Number of teeth missing was obtained from self-reporting questionnaire and confirmed by trained interviewers. Participants were diagnosed as “dementia”, “mild cognitive impairment (MCI)”, or “cognitive normal” by neurologists using DSM-IV and Petersen criteria. Multivariate logistic regression model was applied to examine the association between number of teeth missing and cognitive function.

**Results:**

The study participants had an average of 10.2 teeth lost. Individuals with dementia lost 18.7 teeth on average, much higher than those with MCI (11.8) and cognitive normal (9.3) (p<0.001). After adjusted for sex, age, education year, living alone, body mass index, cigarette smoking, alcohol drinking, anxiety, depression, heart disease, hypertension, diabetes, and APOE-ε4, tooth loss of >16 were significantly associated with dementia with an OR of 1.56 (95%CI 1.12-2.18).

**Conclusion:**

Having over 16 missing teeth was associated with severe cognitive impairment among Chinese older adults. Poor oral health might be considered as a related factor of neurodegenerative symptom among older Chinese population.

## Introduction

Dementia has a significant impact on individual’s disability and mortality, and it also diminishes quality of life for both patients and their caregivers. Many developing countries and regions, such as China, India, and Latin American, are facing a rapid increase in the numbers and proportion of older adults[1]. Similar to its western counterparts, the prevalence of dementia in China among older people is around 4–7% and the age specific prevalence of Alzheimer’s disease (AD) roughly doubles at 5-year intervals[2,3]. It is estimated that there were over 9 million people with dementia in China in 2010 and this number is expected to increase rapidly in the near future[4].

Dementia, especially sporadic, late-onset AD, is likely due to a complex interaction of environmental, vascular and genetic risk factors. However, the attributable risk associated with each of the vascular or environmental risk factors for AD does not exceed 15%, and approximately half of the risk for AD remains unexplained[5]. Thus, a search for additional, potentially causal risk factors is warranted.

Oral health has been drawn more and more attention on its relationship with cognitive function. In many developed countries, evidence from observational studies suggests that oral heal disorders may be associated with cognitive impairment in community-dwelling older adults[6,7,8,9,10,11,12]. However, such kind of studies has seldom been conducted in developing countries, such as China. In China, many important factors contributing to oral health and cognitive impairment are distinctively different from developed countries. These factors may include accessing to dental care, health care system, nutrition intake, and exposure to environmental hazard[8,13,14].

The aim of this community-based study was to examine the relationship between tooth loss and cognitive function among a sample of older Chinese population, in which the cognitive function of each participant was determined by neurologists through a series of neuropsychological tests.

## Methods

### Ethics Statement

This study was approved by the Medical Ethics Committee of Huashan Hospital, Fudan University, Shanghai, China. A written informed consent was obtained from all of the participants and/or their legal guardian.

### Study site and subjects recruitment

Study subjects were recruited from the Shanghai Aging Study. The Shanghai Aging Study aimed to investigate the prevalence of dementia and mild cognitive impairment (MCI) among older adults residing in the Jing'ansi community of Shanghai[15]. During January 2010 to December 2012, the Shanghai Aging Study enrolled 3,836 out of 5,138 residents (excluding those residing in a nursing home or other institution) aged 60 or older in this community, and who were willing to participate and met the inclusion criteria (i.e., age 60 and above and a registered permanent resident in the community). Potential participants were excluded if they 1) showed severe schizophrenia or mental retardation on their medical record; or 2) had severe problems of vision, hearing, or speaking, and were not able to participate in the neuropsychological evaluation.

### Demographic factors and medical history

The participants were interviewed face-to-face by trained research nurses and neurologists to elicit information on the participant’s demographic characteristics, including the patient’s birth date, gender and education. The body mass index (BMI) was calculated as a person’s weight in kilograms (kg) divided by their height in meters (m) squared. BMI was categorized into underweight (<18.5), normal (18.5–24.9) and overweight (>25)[16]. Other lifestyle factors, such as living alone, household income (<500RMB/month; 500-1200RMB/month, and >1200RMB/month) and cigarette smoking and alcohol drinking habits were obtained. Cigarette smoking was defined as regular cigarette smoking for at least 1 year. Alcohol drinking was defined as regular drinking of Chinese alcohol, rice wine, red wine, or beer for at least 1 year. Participants were asked about their medical histories, as defined by physician-diagnosed hypertension, diabetes, and heart disease (including coronary artery disease and arrhythmia) and confirmed these medical histories from the participants’ medical records.

### Neurological and neuropsychological assessments

Neurologists were assigned to administer the Center for Epidemiologic Studies Depression Scale (CESD) and the Zung Self-Rating Anxiety Scale (ZSAS) for each participant[17,18,19]. These widely used instruments were used to assess whether each participant met the criteria of having a major depressive or anxiety episode within the past week. Anxiety and depression were determined if ZSAS >44 and CESD > = 16. Neurologists also administered the Clinical Dementia Rating (CDR) [20,21] and Brody Activity of Daily Living (ADL)[22] scale to obtain information on cognitive complaints and activities of daily living.

Based on Chinese culture, we translated, adapted and normed neuropsychological tests, which cover domains of global cognition, executive function, spatial construction function, memory, language, and attention, from western countries. Tests used in the study were: 1) Mini Mental State Examination; 2) Conflicting Instructions Task (Go/No Go Task); 3) Stick Test; 4) Modified Common Objects Sorting Test; 5) Auditory Verbal Learning Test; 6) Modified Fuld Object Memory Evaluation; Trail-making test A&B; 8) RMB (Chinese currency) test. Normative data and detail description of these tests were reported elsewhere[15,23]. The neuropsychological tests were administered by study psychometrists according to the education level of each participant. All tests were conducted in Chinese within 90 minutes.

After each clinical assessment, four study neurologists and one neuropsychologist reviewed the functional, medical, neurological, psychiatric, and neuropsychological data and reached a consensus regarding the presence or absence of dementia using DSM-IV criteria[24]. MCI was defined according to the Petersen’s criteria[25].

### Measurement of oral health

Oral health was assessed using a self-administered questionnaire with questions about the number of teeth missing and the medical history of oral health diseases and problems. The number of teeth missing (including third molars) of each participant was observed, counted and confirmed by the interviewers. According to quartiles of the data distribution, the number of teeth missing was categorized into the following categories: 1) 0 to 3 teeth, 2) 4 to 6 teeth, 2) 7 to 16 teeth, and 4) more than 16 teeth lost.

### APOE genotype assessment

DNA was extracted from blood or saliva, collected from the study participants. Apolipoprotein E (APOE) genotyping was conducted by the Taqman SNP method[26]. The presence of at least one ε4 allele was treated as being APOE-ε4 positive.

### Statistical analysis

The continuous variables were expressed as the mean and standard deviation (SD), and the categorical variables were expressed as frequencies (%). The Student t-test, analysis of variance (ANOVA), Pearson Chi-squared test and Cochran Mantel Haenszel Chi-squared were used to compare the continuous variables and categorical variables. Three logistic regression models were used to detect the association between the number of teeth missing and different clinical cognitive diagnosis. Risk was presented as odds ratio (OR). Model 1 was a univariate model. Model 2 and 3 were multivariate models. Model 2 adjusted confounding variables of sex, age, and education year. Model 3 further adjusted confounding variables, such as social economic, life style (smoking, drinking and living status), BMI, medical history (anxiety, depression, heart disease, hypertension and diabetes) and APOE. All of the p-values and 95% confidence intervals (CIs) were estimated in a two-tailed manner. Differences were considered to be statistically significant at p<0.05. The data were analyzed using SAS 9.3 (SAS Institute Inc., Cary, NC, USA).

## Results

The current study included 3,063 community older adults aged 60 or above who had both cognitive assessment and oral health information from the Shanghai Aging Study. Among these 3,063 participants, 1,399 (45.7%) were men. The mean age was 71.3 (SD 8.2) and mean years of education was 11.7 (SD 4.2). Five hundred and fifty-four participants (18.1%) were diagnosed as MCI. One hundred and twenty participants (3.9%) were diagnosed as dementia, according to their performance of neuropsychological tests and ADL. The following characteristics were found to be significantly different for the participants across three groups (cognitive normal, MCI, and dementia): age, education duration, household income, living status, smoking, prevalence of heart disease, hypertension, diabetes, and depression, score of MMSE and ADL. Participants with dementia had oldest age (mean 80.9, SD 7.4), fewest years of education (mean 7.6, SD 5.9), lowest MMSE score (mean 16.9, SD 5.0), and highest ADL score (mean 35.3, SD 17.5) among 3 groups. Subjects with heart disease and hypertension had higher prevalence of cognitive impairment (Table 1).

**Table 1:** Demographic, social economic and medical history of the participants with different clinical cognitive diagnosis.

	Cognitive Normal N = 2389	MCI N = 554	Dementia N = 120	P value*
Sex				
male	1110 (79.3%)	246 (17.6%)	43 (3.1%)	0.06
Female	1279 (76.9%)	308 (18.5%)	77 (4.6%)	
Age, years, mean(SD)	70.0(7.7)	74.8(8.4)	80.9(7.4)	<0.001
Education, years, mean(SD)	12.3(3.8)	10.0(4.7)	7.6(5.9)	<0.001
Body mass index, n(%)				
Under weight	80 (70.2%)	24 (21.1%)	10 (8.8%)	0.061
Normal	959 (78.2%)	224 (18.3%)	44 (3.6%)	
Over weight	1344 (78.6%)	302 (17.7%)	65 (3.8%)	
Monthly household income, n(%)				
<500 RMB	15 (53.6%)	12 (42.9%)	1 (3.6%)	<0.001
500–1200 RMB	23 (56.1%)	14 (34.1%)	4 (9.8%)	
>1200 RMB	2344 (78.6%)	525 (17.6%)	114 (3.8%)	
Living alone, n(%)				0.025
Yes	202 (72.4%)	67 (24.0%)	10 (3.6%)	
No	2183 (78.6%)	485 (17.5%)	110 (4.0%)	
Cigarette smoking, n(%)				0.041
Yes	244 (76.5%)	69 (21.6%)	6 (1.9%)	
No	2138 (78.2%)	483 (17.7%)	114 (4.2%)	
Alcohol drinking, n(%)				0.077
Yes	195 (81.3%)	42 (17.5%)	3 (1.3%)	
No	2194 (77.7%)	512 (18.1%)	117 (4.1%)	
Heart disease, n(%)				<0.001
Yes	281 (68.4%)	105 (25.5%)	25 (6.1%)	
No	2101 (79.5%)	448 (16.9%)	95 (3.6%)	
Hypertension				<0.001
Yes	1255 (74.8%)	338 (20.2%)	84 (5.0%)	
No	1134 (81.8%)	216 (15.6%)	36 (2.6%)	
Diabetes				0.018
Yes	314 (72.9%)	98 (22.7%)	19 (4.4%)	
No	2074 (78.8%)	456 (17.3%)	101 (3.8%)	
MMSE, mean(SD)	28.5(1.7)	26.4(2.9)	16.9(5.0)	<0.001
Anxiety, n(%)				0.057
Yes	46 (66.7%)	18 (26.1%)	5 (7.2%)	
No	2338 (78.3%)	533 (17.9%)	114 (3.8%)	
Depression, n(%)				<0.001
Yes	365 (70.2%)	128 (24.6%)	27 (5.2%)	
No	2018 (79.6%)	423 (16.7%)	93 (3.7%)	
ADL, mean(SD)	20.5(3.0)	22.2(6.6)	35.3(17.5)	<0.001
APOE-ε4 allele positive, n(%)				0.451
Yes	20 (76.9%)	5 (19.2%)	1 (3.8%)	
No	2369 (78.0%)	549 (18.1%)	119 (3.9%)	

Abbreviation: SD: standard deviation; MCI: mild cognitive impairment; MMSE: Chinese Mini-Mental State examination; ADL: Activity of Daily Living scale; APOE: Apolipoprotein E

*P value is for the comparison among three groups of cognitive normal, MCI and dementia.

Table 2 showed that the mean number of teeth missing was 10.2 (SD = 9.7) for all the participants. Participants with dementia had a significantly higher number of teeth missing (mean 18.7) than those with MCI (mean 11.8) and cognitive normal (mean 9.3) (p<0.001). Sixty percent of participants with dementia had lost more than 16 teeth. Number of teeth missing was associated with cognitive impairment. Subjects loss more than 16 teeth reported higher prevalence of dementia (p<0.001).

**Table 2:** Number of teeth missing among participants with different clinical cognitive diagnosis.

	All N = 3063	Cognitive Normal N = 2389	MCI N = 554	Dementia N = 120	P value
Number of teeth missing, mean (SD)	10.2 (9.7)	9.3 (9.3)	11.8 (9.9)	18.7 (11.0)	<0.001
0–3, n (%)		823 (84.8%)	131 (13.5%)	17 (1.8%)	<0.001
4–6, n (%)		540 (80.2%)	120 (17.8%)	13 (1.9%)	
7–16, n (%)		528 (77.3%)	137 (20.1%)	18 (2.6%)	
>16, n (%)		498 (67.7%)	166 (22.6%)	72 (9.8%)	

Abbreviation: SD: standard deviation; MCI: mild cognitive impairment.

P value is for the comparison among three groups of cognitive normal, MCI and dementia.

ORs for number of teeth missing with different clinical cognitive diagnosis were presented in Table 3. Model 1, as the univariate model, showed that participants who lost >16 teeth had an OR for dementia of 3.65 (95%CI 2.75–4.86), and had an OR for MCI of 1.42 (95%CI 1.21–1.66). After adjusted for sex, age and education year, the risk of dementia was high for >16 tooth loss, with an OR of 1.59 (95%CI 1.15–2.20) (model 2). Model 3 demonstrated that, after adjusted for sex, age, education year, living alone, BMI, cigarette smoking, alcohol drinking, anxiety, depression, heart disease, hypertension, diabetes, and APOE-ε4, tooth loss of >16 were significantly associated with dementia with an OR of 1.56 (95%CI 1.12–2.18). The association between tooth loss of >16 and MCI was no longer significant after adjusting the confounders (model 2 and model 3).

**Table 3:** Odd ratios for number of teeth missing among participants with different clinical cognitive diagnosis.

	Model 1		Model 2		Model 3	
	Dementia *vs* Normal	MCI *vs* normal	Dementia *vs* Normal	MCI *vs* normal	Dementia *vs* Normal	MCI *vs* normal
Variable	OR (95%CI)	OR (95%CI)	OR (95%CI)	OR (95%CI)	OR (95%CI)	OR (95%CI)
N of tooth loss						
0–3	1.00 (reference)	1.00 (reference)	1.00 (reference)	1.00 (reference)	1.00 (reference)	1.00 (reference)
4–6	0.61 (0.39–0.95)	0.94 (0.80–1.12)	0.88 (0.55–1.40)	1.10 (0.92–1.32)	0.92 (0.57–1.47)	1.11 (0.93–1.33)
7–16	0.86 (0.58–1.29)	1.10 (0.94–1.30)	0.78 (0.52–1.19)	1.05 (0.89–1.24)	0.77 (0.50–1.17)	1.05 (0.88–1.25)
>16	3.65 (2.75–4.86)	1.42 (1.21–1.66)	1.59 (1.15–2.20)	0.95 (0.80–1.14)	1.56 (1.12–2.18)	0.95 (0.80–1.14)

Model 1: Univariate analysis

Model 2: multivariate logistic regression model, adjusted for sex, age and education year;

Model 3: multivariate logistic regression model, adjusted for sex, age, education year, living alone, overweight, cigarette smoking, alcohol drinking, anxiety, depression, heart disease, hypertension, diabetes, and Apolipoprotein E-ε4.

Abbreviation: MCI: mild cognitive impairment; OR: odds ratio; CI: confidence interval.

## Discussion

This cross-sectional study examined the association between tooth loss and clinical diagnosis of cognitive impairment in a community-based older population in Shanghai, China. Having more than 16 missing teeth was found to be positively associated with dementia after controlling for confounders of socio-demographic characteristics, heath behaviors, and medical conditions. This is the first epidemiological study reporting the data in older Chinese population.

The association between oral health and cognitive function is biologically plausible. Potential mechanisms include inflammatory mediators produced in response to periodontal pathogens[27,28,29,30,31], which produce chronic systemic inflammation and neuropathology; increasing risk of stroke and cerebrovascular disease in those with periodontal disease; dissemination of oral gram negative bacteria to the brain via a transient bacteremia[32,33,34,35]; low intake of nutrients, which could cause tooth loss due to vitamins B deficiency[36] and mercury released from dental amalgam[37]. Oral bacteria also may spread to the brain via neuronal pathways. Riviere and colleagues suggested that oral bacteria may reach the brain via branches of the trigeminal nerve. In their postmortem examination of brain tissues, the authors detected antigens of oral treponemes more often in the sample from participants with Alzheimer’s disease (14 out of the 16 participants) than in the ones from the control (4 out of 18)[38].

Self reported number of teeth was widely used as a measure in national epidemiological surveys that examines the relationship between oral health and cognitive function[39–44]. Previous studies conducted in the United States[45] and Japan[46] indicated that self-reported number of teeth had high level of agreement with the ones collected from clinical examination data (Pearson correlation coefficient: r = 0.97). The mean number of teeth missing was 10.7 among our study population which was comparable to other surveys conducted in China in which the mean number of teeth missing was about 10 for old adults aged from 60 to 74 [47, 48]. Additionally, to avoid the recall bias of the participants, especially those with cognitive impairment, the number of teeth missing of each participant was observed, counted and confirmed by the interviewers. Thus, the information obtained on tooth loss in our study should be reliable to a large extent.

We use a total of 32 teeth (include the third molar) rather than a total of 28 teeth (exclusion the third molar) in the calculation of teeth missing is due to the following reasons: 1) When study number of missing teeth or edentulism, it is common for researchers to include third molar. 2) In addition, it is uncommon for Chinese to extract their wisdom teeth. 3) Finally, the results did not differ when we use either approach. Thus, the inclusion of the third molar has it validity.

As a cross-sectional study, our finding on the significant association between tooth loss and dementia is consistent with the previous observational studies. Fewer teeth was positively associated with cognitive impairment (OR 1.26; 95%CI: 1.00–1.59) in 686 adults aged 65 and older in a community based cross-sectional study in Korea[49]. Persons with multiple tooth loss had significantly higher odds of cognitive impairment (OR 2.10, 95%CI: 1.35–3.25) in a nationally representative survey of 557 Swedish population aged 77 and older[50]. The HARMONY study in Sweden indicated that history of tooth loss before age 35 was a significant risk factor of AD from the case-control analysis (OR = 1.49, 95%CI:1.14–1.95) and the co-twin analysis (OR = 3.60, 95%CI: 1.34–9.70)[42]. Two cross-sectional studies on older adults in Japan revealed that lower number of tooth remain(0–10) was a significant independent risk factor of cognitive impairment.[12, 51]. The population-based Study of Health in Pomerania, Germany which comprises 1336 subjects (60–79 years) found that, in the fully adjusted models, tooth loss was associated with low MMSE score in females but not in males[52]. Fewer number of teeth was positively associated with cognitive impairment in the Health, Aging and Body Composition (Health ABC) Study[9] and the Hispanic Established Populations for Epidemiologic Studies of the Elderly[6].

Number of teeth missing is a health outcome accumulated across lifespan, but occurring more frequently in later life[53]. Tooth loss might be caused by severe periodontal infection in the past[54], unhealthy dietary intake, poor oral hygiene care, and lack of dental care. Given tooth loss is very common among older adults, it is possible the relationship between dementia and tooth loss becomes prominent among those who have significant tooth loss, which means lost >16 teeth in our study. Future studies are warranted to further examine the mechanism the relationship between number of teeth missing and dementia.

In the present study, other oral conditions, including caries, periodontitis, and their histories were collected using self-reported questionnaire but may not be reliable. Thus, we did not include the caries and periodontal diseases variables and just focus on the number of teeth missing in this study. Long-term histories on oral health would also be helpful to clarify the association between oral health and cognition function decline. However, most of the respondents were not able to remember the exact time of the onset of the oral health problems and diseases and very few had dental records available in the present study. Compared with their Western counterparts, Chinese have much fewer dental examinations in their life course. The information on the diagnosis date of caries and periodontits in medical records was also not reliable.

Strengths of the current study were that the sample was relatively large and response rates were also reasonable for a study of this scale. Meanwhile, the measurement of cognitive function was reliable because the clinical diagnosis was conducted by neurologists of top institution of neurology in China. One limitation of our study is cross-sectional nature of the data, and thus the direction of causation can hardly be concluded. The follow-up data of the Shanghai Aging Study would be helpful to clarify this issue. Another limitation is that oral health information was not collected by dental professionals. However, the number of teeth missing was observed and confirmed by trained interviewers, which should increase the validity of the data we collected. It is also worth noting that the question on number of tooth remaining is less susceptible to recall error than many other questions that require recall for past events. In addition, although we have adjusted some potential confounding variables in the present study, we still could not exclude the possible influence of unmeasured confounding factors. Lastly, our study site is located in the center of urban Shanghai and the residents have higher level of education than most of the communities in China. Therefore, the study findings may not be generalizable to the whole older population in China.

In conclusion, the number of teeth missing was significantly associated with severe cognitive impairment. Poor oral health might be considered as a related factor of neurodegenerative symptom among older Chinese population. To further elucidate the role of oral health on cognition, prospective studies with larger sample size are needed by inclusion of both clinical diagnosis of cognition and oral health and information on inflammatory biomarkers.
